# Seleno-Amino Acids in Vegetables: A Review of Their Forms and Metabolism

**DOI:** 10.3389/fpls.2022.804368

**Published:** 2022-02-02

**Authors:** Jiangtao Hu, Zheng Wang, Li Zhang, Jie Peng, Tao Huang, Xiao Yang, Byoung Ryong Jeong, Qichang Yang

**Affiliations:** ^1^Institute of Urban Agriculture, Chinese Academy of Agricultural Sciences, Chengdu National Agricultural Science and Technology Center, Chengdu, China; ^2^Division of Applied Life Science (BK21 Four), Department of Horticulture, Graduate School of Gyeongsang National University, Jinju, South Korea; ^3^Institute of Agriculture and Life Science, Gyeongsang National University, Jinju, South Korea; ^4^Research Institute of Life Science, Gyeongsang National University, Jinju, South Korea

**Keywords:** plant factory, precise control, selenium metabolism, seleno-amino acids, vegetables, mushrooms

## Abstract

Seleno-amino acids are safe, health-promoting compounds for humans. Numerous studies have focused on the forms and metabolism of seleno-amino acids in vegetables. Based on research progress on seleno-amino acids, we provide insights into the production of selenium-enriched vegetables with high seleno-amino acids contents. To ensure safe and effective intake of selenium, several issues need to be addressed, including (1) how to improve the accumulation of seleno-amino acids and (2) how to control the total selenium and seleno-amino acids contents in vegetables. The combined use of plant factories with artificial lighting and multiple analytical technologies may help to resolve these issues. Moreover, we propose a Precise Control of Selenium Content production system, which has the potential to produce vegetables with specified amounts of selenium and high proportions of seleno-amino acids.

## Introduction

Selenium (Se) is an essential trace element for human health. For adults (≥18 years), the recommended nutrient intake (RNI) of Se for both genders in Chinese populations is 60 μg per day (National Health Commission, 2018). Appropriate Se supplementation has been reported to exert anti-viral effects, reduce the levels of thyroid autoantibodies, and decrease the risk of cardiovascular disease, type 2 diabetes, and Keshan and Kashin-Beck diseases (Stranges et al., 2007; Rees et al., 2013; Wichman et al., 2016; Liu et al., 2018; Muzembo et al., 2019; Zhang et al., 2019a). However, 500 to 1,000 million people worldwide consume less or more than the recommended levels of Se (Shreenath et al., 2018). Both excessive and deficient intake of Se are associated with health risks. Dietary deficiency of Se in humans is associated with an increased risk of death, hypoimmunity, and cognitive decline (Rayman, 2012), while excess Se supplementation may cause toxicity as Se is involved in the generation of reactive oxygen species and oxidation of thiol compounds, which can lead to oxidative damage in cells (Rayman et al., 2018; Pyrzynska and Sentkowska, 2021). Therefore, a suitable dietary source of Se supplementation containing appropriate levels of this element could be beneficial for human health.

The main sources of Se in the diet are meats and cereals, which contribute more than 50% of the total dietary Se intake in the British and Chinese populations (Gao et al., 2011; Rayman, 2012; Yu et al., 2015). Vegetables and fruits only contribute around 7% of the total dietary Se intake in the British and Chinese populations (Rayman, 2012; Yu et al., 2015). Indeed, vegetables are recognized as relatively weak sources of dietary Se, as they generally contain less than 0.1 μg g^−1^ fresh matter (FM) of Se (Rayman, 2012). However, some species of vegetables are Se accumulators, such as *Brassicaceae* vegetables, garlic, and onions (Finley, 2005). For instance, garlic can accumulate more than 1,300 μg g^−1^ dry matter (DM) of Se, with 73% in the form of γ-glutamyl-Se-methylselenocysteine (γ-Glu-MeSeCys; Ip et al., 2000). Considering their relatively high consumption, rapid growth (less than 30 days for harvest of leafy greens), and ability to accumulate Se, vegetables hold great potential as Se-fortified sources for dietary Se intake.

Seleno-amino acids (Se-AAs) are organic forms of Se and are thus thought to be ideal chemical forms for Se supplementation. Organic forms of Se have been reported to be lower in toxicity compared to inorganic Se. Vinceti et al. (2017a) reported that consumption of approximately 260 μg per day organic Se led to toxic effects; the corresponding value for inorganic Se was 16 μg per day for humans. Vinceti et al. (2017b, 2018) reported that overexposure to inorganic Se was associated with Alzheimer’s dementia, neurodegenerative diseases, amyotrophic lateral sclerosis, and Parkinson’s disease. Moreover, dietary organic Se has high bioavailability because most of them can reach the systemic circulation from the gastrointestinal tract and can promote its action in the exposed organism. Previous studies reported that 70–90% of Se in Se-enriched plant foods could be transformed into organic forms and distribute and function in human organs and tissues (Pyrzynska and Sentkowska, 2021). Among the various Se-AAs, selenomethionine (SeMet) has the highest bioavailability of more than 90%, which is 1.5 times higher than that of selenite (Burk and Hill, 2015; Xie et al., 2021). *In vitro* simulated gastrointestinal digestion studies suggested SeMet is the major form of bioaccessible Se released from food matrices (Bhatia et al., 2013; Wang et al., 2013; do Nascimento da Silva et al., 2017). Overall, due their abundance in vegetables, low toxicity, and high bioavailability, Se-AAs are thought to be valuable forms of dietary Se supplements. In this regard, it is of interest to develop Se-enriched vegetables with high-Se-AA contents to ensure safe, sufficient intake of Se.

In this review, we critically explore the literature related to the forms of Se-AAs and Se concentrations of different vegetables, the factors that affect Se speciation, and the metabolism of Se-AAs in plant species. This comprehensive review may aid the innovation and development of Se-enriched vegetables with high-Se-AA contents in agricultural practice.

## Vegetables as Dietary Supplements of Se

### Se-AA Forms and Concentrations in Different Species of Vegetables

**Figure 1:**
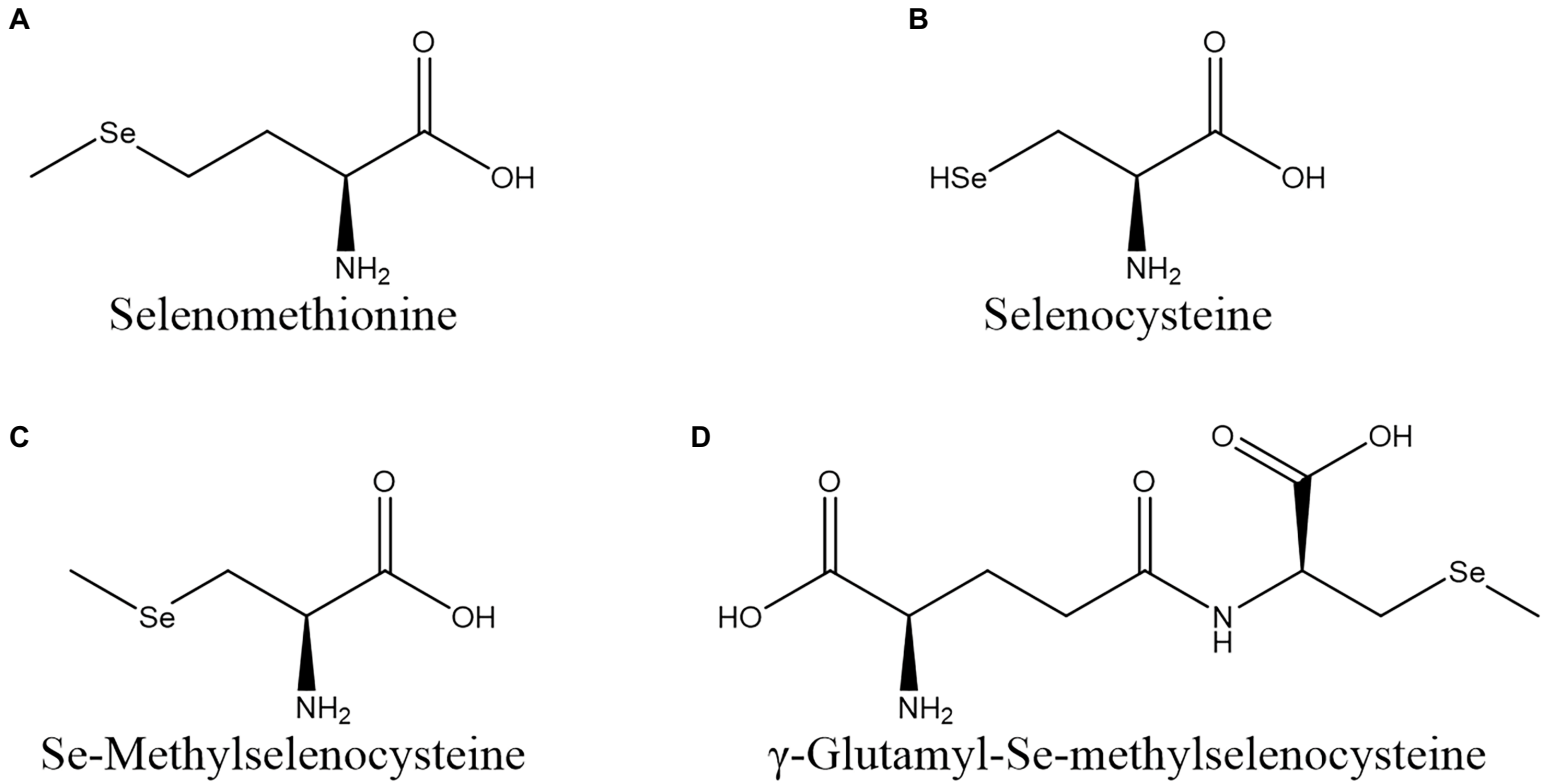
Structures of seleno-amino acids commonly found in vegetables. **(A)** SeMet; **(B)** SeCys; **(C)** MeSeCys; and **(D)** γ-Glu-MeSeCys.

Vegetables can biotransform inorganic Se into Se-AAs, such as SeMet, selenocysteine (SeCys), Se-methylselenocysteine (MeSeCys), and γ-Glu-MeSeCys (Figure 1). These Se-AAs can be almost completely absorbed by human organs and are beneficial for human health.

We reviewed the concentrations of total Se and the main Se-AAs in the edible parts of vegetables and mushrooms biofortified with Se (Table 1). The main Se-AAs in vegetables exhibit species-specific patterns, regardless of the Se source and application method. Vegetables belonging to the *Brassicaceae* and *Liliaceae* families predominantly accumulate MeSeCys, while other vegetables tend to accumulate SeMet. For vegetables that mainly accumulate MeSeCys, the highest total Se concentration was found in broccoli (1798.4 ± 58.8 μg g^−1^ DM) and the highest MeSeCys concentration was found in broccoli sprout (168.9 ± 19.0 μg g^−1^ DM). For those mainly accumulate SeMet, the highest total Se concentration was found in lettuce (602.0 ± 6.0 μg g^−1^ DM) and the highest SeMet concentration was found in Lion’s Mane mushroom (55.1 ± 11.1 μg g^−1^ DM). Studies reported that MeSeCys can be quickly converted into methyl selenol, which exerts putative anti-cancer activity (Ip et al., 2000). Therefore, vegetables from the *Brassicaceae* and *Liliaceae* families may represent suitable dietary sources for Se supplementation.

**Table 1:** Concentrations of total selenium and the main seleno-amino acids in the edible parts of vegetables and mushrooms biofortified with selenium.

Family	Common name	Se source and dose	Application method	Total Se concentration (μg g^−1^)	Main Se-AAs and concentration (μg g^−1^)	Reference
*Agaricaceae*	Mushroom	NG	NG	9.15, DM	SeCys, 5.73, DM	Hu et al., 2021b
*Agaricaceae*	Mushroom	Nanoparticles, 10 μg g^−1^ substrate	Substrate application	About 10, DM	SeMet, 2.01, DM	Hu et al., 2021a
*Agaricaceae*	Mushroom	Selenite or selenate, ≤5 μg g^−1^ substrate	Substrate application	5.32 or 3.36, DM	SeMet, NG	Zhou et al., 2018
*Agaricaceae*	Mushroom	Selenite, 6.4 μM	Substrate application	23.1–31.0, DM	SeMet, 17.1–23.1, DM	Milovanovic et al., 2019
*Agaricaceae*	Mushroom	Selenite or selenate, 1.2 mM or 26.5 μM	Substrate application	111.8 or 45, DM	SeMet, 55.1 or 24.3, DM	Hu et al., 2020
*Agaricaceae*	Mushroom	Selenite, 5 μg g^−1^ substrate	Substrate application	59.6, DM	SeMet, 33.9, DM	Dong et al., 2021
*Apiaceae*	Carrot	Selenite or selenate, ≤0.5 mM	Foliar spray	1.5 or 2.2, DM	SeMet, 0.43 or 0.37, DM	Kápolna et al., 2009
*Brassicaceae*	Broccoli, cauliflower, green cabbage, Chinese cabbage, kale, and Brussels sprouts	Selenate, 50 μM	Hydroponic application	160 on average, DM	SeMeCys, 80 on average, DM	Ávila et al., 2014
*Brassicaceae*	Broccoli	Selenate, 50 g ha^−1^	Foliar spray	521–955, FM	SeMet and SeMeCys, 52–120, DM	Šindelářová et al., 2015
*Brassicaceae*	Broccoli	Selenate, 20 μM	Hydroponic application	801.2–1798.4, DM	SeMet and SeMeCys, about 0.5 μM g^−1^ FM	Ramos et al., 2011
*Brassicaceae*	Cabbage	Selenate, 0.1 mM or 2.6 μM	Foliar spray or soil application	0.96 or 4.80, DM	SeMet, 0.18 or 2.52, DM	Mechora et al., 2012
*Brassicaceae*	Pak choi	Selenite, 10 μM	Hydroponic application	2.22, FM	SeMeCys, 0.61, FM	Yu et al., 2019
*Brassicaceae*	Pak choi	Selenate, 10 μM	Hydroponic application	42.17, FM	SeMet, 6.46 FM	Yu et al., 2019
*Brassicaceae*	Pak choi, kale, and broccoli sprouts	Selenate, <0.64 mM	Sand culture and nutrient supplement	155.9–467.1, DM	SeMeCys, 57.4–168.9, DM	Thosaikham et al., 2014
*Brassicaceae*	Radish	Nanoparticles, 12.7 μM	Hydroponic application	144, FM	SeMeCys, 43, FM	Palomo-Siguero et al., 2015
*Brassicaceae*	Radish	Selenate, ≤10 μM	Foliar spraying	120, DM	SeMeCys, 33, DM	Schiavon et al., 2016
*Brassicaceae*	White cabbage, broccoli, mustard, and rye sprouts	Selenium dioxide, 90.1 μM	Hydroponic application	53.3–400.0, DM	SeMet and SeMeCys, NG	Piekarska et al., 2014
*Compositae*	Lettuce	Selenite or selenate, ≤40 μM	Hydroponic application	50.8 or 602.0, DM	SeMet, 6.9 or 25.2, DM	do Nascimento da Silva et al., 2017
*Leguminosae*	Chickpea	Selenite or selenate, ≤40 g ha^−1^	Soil application	0.70 or 2.92, DM	SeMet, 0.46 or 1.52, DM	Poblaciones et al., 2014
*Leguminosae*	Lentil and soy sprouts	Selenite and selenate (1:1), ≤23.1 μM	Hydroponic application	98–284, DM	SeMet, 14.9–29.1, DM	Funes-Collado et al., 2013
*Leguminosae*	Soybean	Selenite, 5 μg g^−1^ soil	Soil application	75, DM	SeMet and SeCys, NG	Chan et al., 2010
*Liliaceae*	Garlic	Nanoparticles, 12.7 μM	Hydroponic application	About 22, DM	SeMeCys, About 16.06, DM	Li et al., 2020
*Liliaceae*	Garlic	NG	NG	1.36, DM	SeMeCys, NG	Kotrebai et al., 2000
*Liliaceae*	Onion	NG	NG	0.14, DM	SeMeCys, NG	Kotrebai et al., 2000
*Liliaceae*	Ramp	NG	NG	0.52, DM	SeMeCys, NG	Kotrebai et al., 2000
*Solanaceae*	Potato	Selenite or selenate, 100 g ha^−1^	Foliar spray	0.78 or 1.22, DM	SeMet, 0.61 or 0.41, DM	Zhang et al., 2019b
*Solanaceae*	Potato	Selenate, 52.7 μM	Foliar spray	1.1, DM	SeMet, 0.33, DM	Cuderman et al., 2008

DM, dry matter; FM, fresh matter; and NG, not given.

### Percentage of Minimum Recommended Daily Allowance and Acceptable Daily Intake Based on Se Accumulation

Dietary intake of Se is largely dependent on the soil levels of Se, which vary in different regions. Daily Se intake is less than 11 μg in the Se-deficient regions of China where Keshan and Kashin-Beck diseases occur (Liu et al., 2018); in contrast, the average Se intake is 550 μg per day in Enshi, China, a Se-rich region (Yuan et al., 2012; Huang et al., 2013). However, it was suggested that an intake of above 400 μg Se per day would lead to chronic toxicity (Winkel et al., 2012; Malagoli et al., 2015). Due to the narrow safe intake range, daily consumption of Se-enriched vegetables should be carefully considered. Therefore, based on the current biofortification methods (Table 1), we calculated the percentage of minimum recommended daily allowance and acceptable daily intake for Se-enriched vegetables and mushrooms (Table 2). The water contents of vegetables used in these calculations were taken from Food Data Central Database (United States Department of Agriculture Food Data Central Database, 2021). We found that 100 g of cabbage, carrot, chickpea, onion, or potato provide less Se than the minimum recommended daily allowance. The same quantities of other vegetables meet the minimum recommended daily allowance, and most exceed the acceptable daily intake. Therefore, it is important to control the Se content of vegetables within a suitable range and to increase the proportion of Se-AAs in vegetables.

**Table 2:** Calculated percentage of minimum recommended daily allowance and acceptable daily intake for selenium-enriched vegetables and mushrooms.

Vegetable	Se content (μg 100 g^−1^ FM)	Percentage of MRDA (%)	Percentage of ADI (%)
Brassica sprouts	2026.7–6072.3	3684.9–11040.5	506.7–1518.1
Broccoli	8012.0–95,500	14567.3–173636.4	2003.0–4496.0
Cabbage	6.7–33.6	12.2–61.1	1.7–8.4
Carrot	14.9–21.8	27.0–39.6	3.7–5.4
Chickpea	2.8–11.7	5.1–21.2	0.7–2.9
Garlic	50.2–811.8	91.2–1476.0	12.5–203.0
Lettuce	269.2–3190.6	489.5–5801.1	67.3–797.7
Lentil and soy sprouts	3038.0–8804.0	5523.6–16007.3	759.5–2201.0
Mushroom	33.6–1118.0	61.1–2032.7	8.4–279.5
Onion	1.4	2.5	0.4
Pak choi	222.0–4217.0	403.6–7667.3	55.5–1054.3
Potato	17.2–26.8	31.2–48.8	4.3–6.7
Radish	480.0–14400.0	872.7–26181.8	120.0–3600.0
Soybean	405.0	736.4	101.3

MRDA, minimum recommended daily allowance and ADI, acceptable daily intake.

## Factors That Affect Se Speciation

Several authors reviewed factors that affect bioavailability of Se (the fraction of Se that is available for absorption by plant) in soil–plant system, including Se speciation, soil property (pH/Eh, metallic oxide, and organic matter and clay contents), plant condition (species, cultivar, and growth stage), climate condition, and agronomic management (tillage management, irrigation, rotation and intercrop management, and fertilizer). Such factors also have impacts on the Se speciation in plants. We summarized the related studies and discussed factors that affect Se speciation, including Se source, agronomic management, and vegetable species and cultivars.

### Se Source

**Figure 2:**
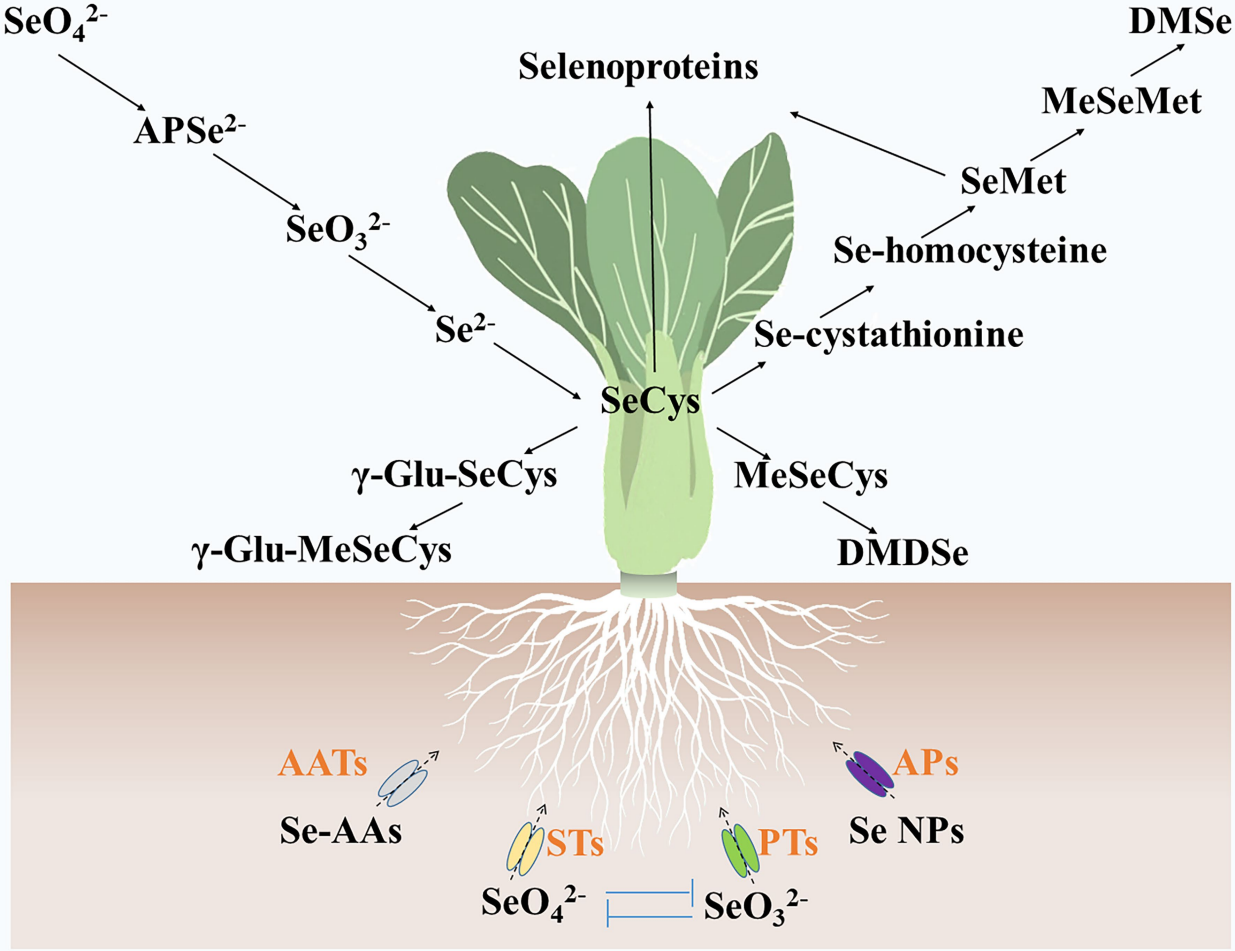
Metabolic fate of selenium in vegetables. Se-AAs, selenate, selenite, and Se nanoparticles are absorbed by vegetables *via* amino acid transporters, sulfate transporters, phosphate transporters, and aquaporins, respectively. Then, these selenium forms were assimilated into various selenium metabolites, such as adenosine phosphoselenate, SeCys, γ-glutamyl-selenocysteine, γ-Glu-MeSeCys, MeSeCys, Se-cystathionine, Se-homocysteine, SeMet, Se-methylselenomethionine, dimethyldiselenide, dimethylselenide, and selenoproteins. AATs, amino acid transporters; APs, aquaporins; PTs, phosphate transporters; and STs, sulfate transporters. SeO_3_^2−^, Selenite; APSe^2−^, adenosine phosphoselenate; SeO_4_^2−^, selenate; Se NPs, Se nanoparticles; γ-Glu-SeCys, γ-glutamyl-selenocysteine; MeSeMet, Se-methylselenomethionine; DMDSe, dimethyldiselenide; and DMSe, dimethylselenide.

Selenite, selenate, Se nanoparticles, and Se-AAs can be absorbed by vegetables (Dinh et al., 2019). They are assimilated into various Se metabolites in the cells of vegetables (Figure 2). Vegetables, such as turnip (Li et al., 2018a), lettuce (Hawrylak-Nowak, 2013), and green pea (Garousi et al., 2017), accumulate more Se contents by supplying selenate as compared with selenite. Studies also showed that selenate treatment resulted in more Se-AA contents in pak choi (Yu et al., 2019) and potato (Zhang et al., 2019b). Selenite and selenate are transported by phosphate transporters and sulfate transporters, respectively (Feist and Parker, 2001; Li et al., 2008; El Mehdawi et al., 2018), and compete with one another when they coexist. Selenite-inhibited selenate uptake and transport in wheat (Li et al., 2008), whereas high concentrations (0.63 μM) of selenate prevented selenite absorption in maize (Longchamp et al., 2013). The Se nanoparticles were found to be transported by aquaporins (Hu et al., 2018; Wang et al., 2020a), and their metabolic fate was similar to that of selenite (Palomo-Siguero et al., 2015; Hu et al., 2018; Wang et al., 2020a). It was suggested that Se-AAs may be transported by amino acid transporters (Kikkert and Berkelaar, 2013). However, vegetables contain dozens of amino acid transporters (Yang et al., 2020), and the specific amino acid transporters that transport Se-AAs have not yet been identified.

#### Inorganic Se as a Se Source for Biofortification

Selenite and selenate are frequently employed as inorganic sources of Se for plant biofortification. Absorbed selenite is rapidly converted into Se-AAs in the roots of wheat (Li et al., 2008) and pak choi (Yu et al., 2019), with only a small proportion of selenite being translocated to the shoots. In contrast, selenate is gradually converted into Se-AAs in the roots, with the majority of selenate being translocated to the shoots.

The Se nanoparticles have recently been proposed as a new type of fertilizer and can be synthesized *via* physical, chemical, or biological processes (Wadhwani et al., 2016; Skalickova et al., 2017) with varied particle sizes, stabilities, and bioavailabilities. Several studies have revealed that Se nanoparticles are oxidized to selenite in plants, implying that their metabolic fate is similar to that of selenite (Palomo-Siguero et al., 2015; Hu et al., 2018; Wang et al., 2020a). However, different plants exhibit distinct patterns of biotransformation of Se nanoparticles. Rice seedlings more efficiently biotransformed Se nanoparticles into Se-AAs than wheat seedlings which were grown in nutrient solutions in greenhouses (Hu et al., 2018; Wang et al., 2020a).

Even though inorganic sources of Se are largely used for vegetable biofortification, they have two disadvantages. Firstly, inorganic Se can only be used in low concentrations, otherwise stunting of root and plant growth, chlorosis, or withering may occur (Terry et al., 2000). Secondly, only some of the inorganic Se is biotransformed into Se-AAs. High proportions of the inorganic forms remain in vegetables (Thosaikham et al., 2014). Therefore, alternative strategies need to be explored to improve the bioavailability of inorganic Se to vegetables and to reduce the proportion of inorganic Se in the edible parts of vegetables.

#### Organic Se as a Se Source for Biofortification

Organic Se is the main form in agricultural soils under different cropping systems in Enshi, China, which accounts for 56–81% of the total Se (Qin et al., 2017). They have distinct availabilities to different plants. Kikkert and Berkelaar (2013) determined that SeMet and SeCys were more readily absorbed by durum wheat and spring canola than inorganic Se. It was also shown that SeMet was more effective than selenate for the production of Se-enriched garlic and Indian mustard (Ogra et al., 2017). On the contrary, inorganic Se was more bioavailable to oilseed rape than Se-AAs (Ebrahimi et al., 2015). In addition, Eich-Greatorex et al. (2007) found no differences in the uptake of organic Se (Se yeast and SeMet) and inorganic Se (selenate) fertilizers in wheat, barley, and oats. These results imply that Se-AAs can be utilized by plants; however, there is limited detailed information on the absorption, translocation, and accumulation of Se-AAs in individual vegetables.

We hypothesize that Se-AAs, particularly water-soluble MeSeCys and methylselenomethionine, can be absorbed directly by plants as several families of amino acids transporters have been identified, including the amino acid transporter family, amino acid-polyamine-choline, mitochondrial carrier family, preprotein and amino acid transporters, and the divalent anion: Na^+^ symporter (Rentsch et al., 2007). In addition, methionine was previously reported to hinder SeMet uptake (Sandholm et al., 1973), implying that SeMet and methionine share the same transporters. Frommer et al. (1993) identified a broad specificity amino acid permease (pAAP1) in *Arabidopsis thaliana* that transported proline *via* a process in which methionine was a strong competitor. Therefore, pAAP1 may be capable of transporting SeMet.

The Se hyperaccumulators, such as *Astragalus* and *Stanleya pinnata* species, are not edible, at least not directly. These plants are rich in MeSeCys or SeMet and hence could be employed as organic Se sources for vegetable biofortification (Bañuelos et al., 2015, 2016; Wu et al., 2015). The use of seed meals derived from Se-enriched mustard and canola was shown to increase the Se content of strawberry fruits (Banuelos and Hanson, 2010). Amendment of the soil with Se-enriched *Stanleya pinnata* led to production of carrots and broccoli with SeMet as the predominant organic Se compound (Bañuelos et al., 2015, 2016). Wang et al. (2018) revealed that amendment of Se-enriched wheat straw and pak choi increased the soil respiration rate and resulted in increased levels of soluble Se, exchangeable Se, and fulvic acid-bound Se, all of which contributed to higher Se bioavailability. Importantly, Se-enriched plants should be pre-incubated in soils to ensure the bioavailability of Se (Stavridou et al., 2011); otherwise, a reduced efficiency of Se uptake and fertilizer recovery would be observed (Ebrahimi et al., 2019). Overall, the Se present in Se-enriched plants is accessible to plants, and thus, Se hyperaccumulators, such as *Astragalus* species, that grow in Se-rich areas have the potential to be employed as natural and green sources of Se; a better understanding of the metabolic fate of MeSeCys and SeMet would help to rationally develop Se resources and produce Se-enriched vegetables with high-Se-AA contents.

### Agronomic Management

Agronomic management has been widely used to regulate the quality of vegetables (Yang et al., 2021). Current studies indicate that the method and timing of application of Se fertilizers, mineral elements in the rhizosphere, and external conditions affect the levels of Se-AAs in various plant species, though only a few studies have investigated these factors in vegetables. However, these results may provide practical guidance for production of Se-enriched vegetables with high-Se-AA contents.

#### Application Method and Timing of Se Fertilizers

The Se fertilizers are generally applied *via* foliar and root application. Foliar application offers the advantages of enhanced utilization efficiency associated with prevention of environmental pollution (Niu et al., 2021). Application of Se at a critical growth stage is essential to ensure higher plant Se-AA contents. Studies have shown that the levels of organic Se were 2-fold higher in rice grains sprayed with 75 g ha^−1^ of selenate or selenite at the full heading stage than plants treated at the late tillering stage (Deng et al., 2017). In wheat, foliar spraying of 20 g ha^−1^ selenate at the pre-filing stage increased organic Se (mainly SeMet) by 5.34% compared to spraying at the pre-flowering stage (Wang et al., 2020b). In blueberry plants, foliar application of 200 g ha^−1^ selenate or selenite during the young fruiting stage resulted in 12.9–16.6% higher organic Se levels than treatment during the coloring stage (Li et al., 2018b).

Even though foliar application has advantages, Yin et al. (2019) found that root application of Se led to 91.2–97.1% higher Se-AA levels in rice than foliar application. However, root application generally adds more Se fertilizer to the soil and water, which leads to concerns related to possible long-term environmental impacts (Tan et al., 2016). Therefore, it is critical to optimize the application methods for different vegetables.

#### Mineral Elements in the Rhizosphere

Duncan et al. (2017) found that nitrogen fertilizers increased the total Se content and the proportion of undefined Se by 60–70% but decreased the major form of organic Se (SeMet) in wheat grains. Additionally, phosphorus and sulfur have been established to compete for selenite and selenate absorption, respectively (Feist and Parker, 2001; Li et al., 2008; El Mehdawi et al., 2018). Interestingly, phosphorus application at 160 μg g^−1^ decreased the total and organic Se contents in the grains of winter wheat, while the proportion of organic Se increased by 7.2–15.1% compared to no phosphorus application (Nie et al., 2020). Although sulfur deficiency enhanced the total Se content of wheat seedlings supplied with 10 μM selenate, the contents of MeSeCys in the shoots and roots decreased by 74.9 and 82.3%, respectively (Huang et al., 2017).

#### External Condition

External conditions, such as climate conditions and soil properties, have influences on the bioavailability of Se through affecting the absorption of Se by vegetables and Se fraction and speciation in the soil (Dinh et al., 2019). Renkema et al. (2012) found that enhanced transpiration at around 50% relative humidity increased Se translocation by up to 6-fold in durum wheat and spring canola. Soil pH governs Se speciation and changes the charges of bioavailable Se (Nakamaru and Altansuvd, 2014; Ponton et al., 2018). SeMet becomes negatively charged as the pH increases from 7 to 10; therefore, SeMet is more bioavailable at high pH (Ponton et al., 2018). Numerous studies have also been conducted to determine the influence of selenobacteria (Se biofortification by certain bacteria in the soil) on the accumulation of Se in plants. Several genera, including Acinetobacter, Bacillus, Enterobacter, Klebsiella, Paenibacillus, Pseudomonas, and Stenotrophomonas, were isolated and identified as Se-tolerant (Acuña et al., 2013; Durán et al., 2014). Among them, Acinetobacter E6.2 produced elevated levels of SeMet (10.0 μg g^−1^ DM) and MeSeCys (3.8 μg g^−1^ DM) without causing oxidative stress (Durán et al., 2015). In addition, co-inoculation of selenobacteria with an arbuscular mycorrhizal fungus further enhanced Se accumulation (Durán et al., 2013, 2016). However, organic matter existed in the soil or organic amendments reduced Se bioavailability to plants by immobilizing Se (Li et al., 2017).

Overall, agronomic management represents a practical method of obtaining Se-enriched vegetables with high-Se-AA contents. Thus, it is important to investigate the optimal application methods and timing of Se fertilization for specific vegetable crops. Phosphorus and sulfur are essential to obtain high contents of Se-AAs, while nitrogen and organic matter should be carefully controlled. Moreover, even though high pH, enhanced transpiration, and selenobacteria have been shown to be beneficial for Se accumulation, the Se speciation and their contents were not determined in the previous studies. Future studies should focus on the precise effects of external conditions on the Se-AAs accumulated by vegetable crops.

### Vegetable Species and Cultivars

Uptake and accumulation of Se are distinct in different plant species. The Se hyperaccumulators generally accumulate 10- to 100-fold higher levels of Se than Se non-accumulators. These plants exhibit higher Se to sulfur ratios (Se preference), organic Se to inorganic Se ratios, shoot to root Se ratios, and source to sink Se ratios (Pilon-Smits, 2017). By taking advantage of elevated selenocysteine methyltransferase (SMT) levels, Se hyperaccumulators produce methylated forms of SeMet and SeCys, rather than integrating these Se-AAs into proteins (Pilon-Smits, 2017). The Se hyperaccumulators are also capable of converting SeCys to elemental Se and alanine (Guignardi and Schiavon, 2017). In addition, they have higher contents of hormones (jasmonic acid, salicylic acid, and ethylene) and enhanced levels of stress-resistance genes, which contribute to Se assimilation and tolerance (Pilon-Smits, 2017). As a result, Se is less hazardous to Se hyperaccumulators, and Se hyperaccumulators generally have higher total Se and Se-AA contents than other plants (Finley, 2005; Wiesner-Reinhold et al., 2017). Similar to Se hyperaccumulators, vegetables from the *Brassicaceae* and *Liliaceae* families accumulate more total Se and higher MeSeCys contents (Table 1). Moreover, vegetables from the *Brassicaceae* family are tolerant to high concentrations of inorganic Se (0.64 mM of selenate by nutrient supplementation, Table 1).

Cultivar also has influence on uptake and accumulation of Se. It was reported that high-Se rice cultivars have been shown to alter the mass flow and activate Se by increasing the rhizospheric pH and secreting organic acids into rhizosphere, which result in improved Se bioavailability (Zhang et al., 2019c). In addition, total contents of SeCys, MeSeCys, and SeMet in broccoli heads were cultivar dependent, ranging from 0.20 to 0.66 μg g^−1^ DM (Šindelářová et al., 2015).

## Metabolism of Se-AAs

### Regulation of Se-AAs in Vegetables

**Figure 3:**
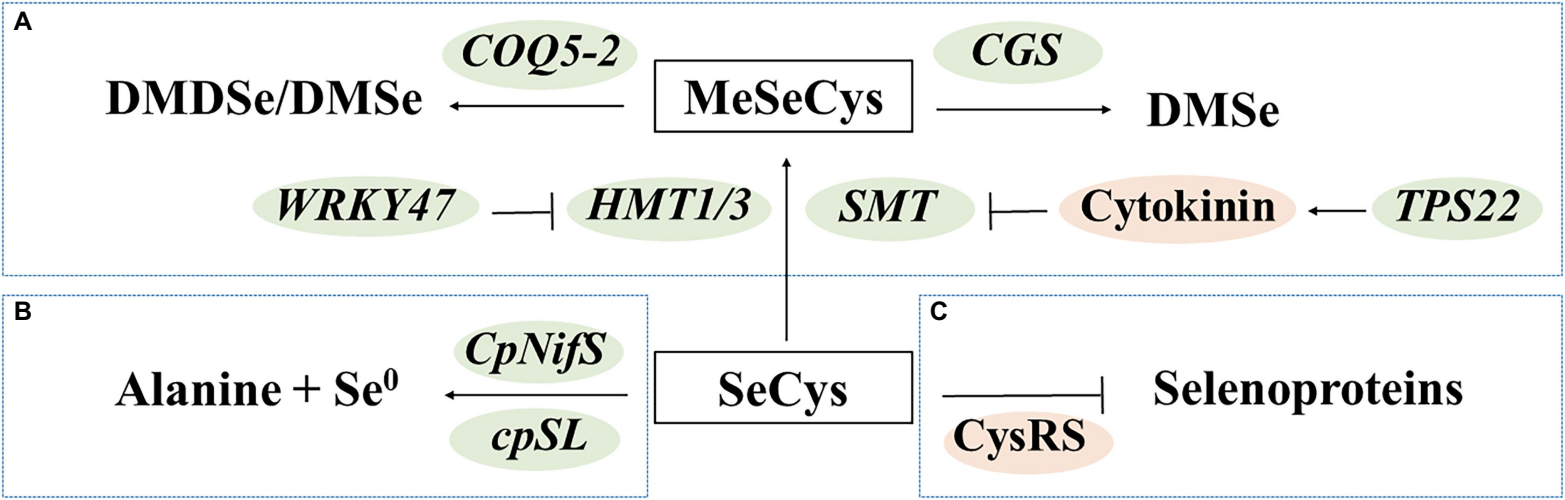
Transgenic approaches to regulate seleno-amino acids in plants. **(A)** Production of methylated and/or volatile forms; **(B)** Decomposition of SeCys; and **(C)** Reduction of misincorporation. DMDSe, dimethyl diselenide; DMSe, dimethyl selenide; MeSeCys, Se-methylselenocysteine; and SeCys, selenocysteine.

Several molecular studies have been conducted to reveal the mechanisms of detoxification in Se hyperaccumulators. The Se hyperaccumulators have been found to mitigate Se toxicity by converting SeCys to methylated and/or volatile forms (Figure 3A), decomposing SeCys (Figure 3B), and preventing SeCys misincorporation (Figure 3C). These findings provide important information for molecular breeding of Se-enriched vegetables with high-Se-AA contents.

The Se hyperaccumulators may exhibit increased expression of Se-assimilation genes (Lima et al., 2018). They also predominantly contain water-soluble and non-protein forms of organic Se compounds, such as MeSeCys (Pilon-Smits, 2017). Researchers have identified *SMT* genes that specifically methylate selenocysteine and homocysteine in vegetables, such as broccoli (Lyi et al., 2005) and Indian mustard (Chen et al., 2019). Overexpression of *SMT* increased the levels of MeSeCys by more than 95.6% (with selenite as the Se source) or 72.4% (with selenate as the Se source) in tomato fruits (Brummell et al., 2011). Apart from the expression levels of *SMT*, the enzyme activity of SMT is also important. Sors et al. (2009) reported that the SMT of a non-accumulator *Astragalus drummondii* lacked activity *in vitro,* while insertion of mutations increased its activity. The *WRKY47* gene and cytokinin have been associated with Se tolerance in *Arabidopsis thaliana*. The *wrky47* mutants of *A. thaliana* are sensitive to Se stress; these mutants exhibit decreased expression of *HMT1* and *HMT3,* which share significant primary sequence homology with *SMT* (Wu et al., 2020). The *tps22* mutants have decreased exogenous levels of cytokinins, which resulted in increased *SMT* expression (Jiang et al., 2018). Moreover, overexpression of the genes encoding cystathionine-γ–synthase (*CGS*) and COQ5 methyltransferase (*COQ5-2*) increased the production of volatile Se compounds, resulting in a lower Se content and decreased toxicity, in Indian mustard and broccoli, respectively (Huysen et al., 2003, 2004; Zhou et al., 2009).

Decomposing SeCys avoids misincorporation of SeCys into proteins. Overexpression of the genes encoding NifS-like protein (*CpNifS*) and selenocysteine lyase (*cpSL*) enhanced conversion of SeCys into alanine and elemental Se in *A. thaliana* and Indian mustard, respectively (Van Hoewyk et al., 2005; Bañuelos et al., 2007). Decreasing misincorporation of SeCys may reduce toxicity and increase the content of Se-AAs. A variant cysteinyl-tRNA synthetase (*CysRS*) that reduced the frequency of SeCys misincorporation was identified in Se hyperaccumulator *Astragalus bisulcatus* (Hoffman et al., 2019).

In the absence of the abilities described above, Se non-accumulators are sensitive to Se supplementation, thus absorb and accumulate less total Se, and have lower Se-AA contents. Therefore, further studies are needed to select and breed vegetable cultivars with Se hyperaccumulator abilities. In addition, other genes involved in the transport and assimilation of inorganic Se have the potential to increase the content of Se-AAs. Researchers found that sulfate transporters (*Sultr*) and ATP sulphurylases (*APS*) play important roles in the accumulation of Se (Schiavon et al., 2015) and that overexpression of the phosphate transport gene (*OsPT8*) improved the Se content of *Nicotiana tabacum* (Song et al., 2017). However, it is a pity that the Se-AA contents were not determined in these studies.

### Future Research Prospects

**Figure 4:**
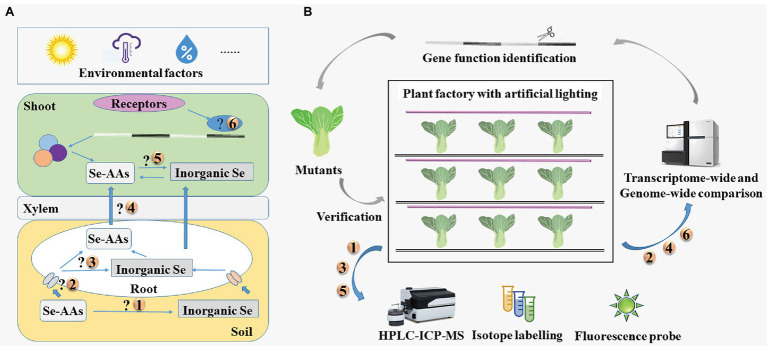
**(A)** Key issues in research of seleno-amino acids in vegetables and **(B)** the proposed strategies to address these issues. HPLC-ICP-MS, high-performance liquid chromatography in conjunction with inductively coupled plasma mass spectrometry; Se-AAs, seleno-amino acids; and Se, selenium.

As described above, Se-AAs can be used as effective, green sources for Se biofortification. However, it is unknown whether Se-AAs are mineralized or directly absorbed by plants, what happens when Se-AAs are allocated into the roots and shoots, and how plants transport Se-AAs from their roots to the shoots (Figure 4A).

Researchers have generally addressed these issues by applying multiple analytical techniques (Figure 4B); however, these analytical techniques have some limitations. Isotope labeling has been used to investigate the metabolic fate and translocation of substances for many years, though the applicability of this approach is limited by the scarcity of isotopically labeled Se speciation (Pedrero and Madrid, 2009; Abdillah et al., 2021). The Se speciation is generally identified by coupling multiple techniques, such as high-performance liquid chromatography in conjunction with inductively coupled plasma mass spectrometry (HPLC-ICP-MS). Fluorescent probes have also been developed for the detection of Se-AAs (Abdillah et al., 2021). However, many probes perform poorly under the physiological conditions of living cells. Therefore, Guo et al. (2020b) designed an Au nanoparticle-based probe for detecting SeCys in plants, such as rice and tea.

The transporters of Se-AAs could be identified using high-throughput sequencing. Plant factories with artificial lighting (PFAL) system provide stable environmental conditions and can be used year-round to grow plants. Amino acid permeases that are responsible for the uptake and transport of Se-AAs could potentially be identified through precise management of environmental conditions and a variety of traits using PFALs. Overall, combined use of multiple technologies would be preferable for determining the mechanisms of uptake and translocation of Se-AAs in vegetables.

## Perspectives

Improving the Se-AA contents of vegetables benefits human health and increases the economic value of vegetables. This review summarized previous research on the forms and metabolism of Se-AAs in vegetables. To produce safe and effective Se-enriched vegetables, a number of important issues still need to be elucidated, such as (1) how can we improve the accumulation of Se-AAs in vegetables and (2) how can we control the ratios of total Se and Se-AA contents of vegetables. Hereafter, we discuss these issues in detail and propose possible solutions.

### Cultivar Selection as an Important Strategy to Improve Accumulation of Se-AAs

Biosynthesis of Se-AAs varies between different cultivars, including wheat (Duncan et al., 2017; Wang et al., 2021), potato (Cuderman et al., 2008), and pear (Bañuelos et al., 2012). It was reported that two rice cultivars supplied with SeMet accumulated significantly different Se contents (Zhang et al., 2006); the high-Se rice cultivar expresses more transporter genes and has a more optimal grain storage capacity than the low-Se rice cultivar (Zhang et al., 2020). In addition, accumulation of Se-AAs can be improved by enhancing their translocation. Amino acid permeases may function as transporters of Se-AAs, as they transport amino acids from the soil into root cells and allocate the Se-AAs from the roots to shoots (Garneau et al., 2018; Guo et al., 2020a). As mentioned above, the majority of Se-AAs accumulated in the roots of wheat and pak choi. The Se-AAs did not appear to translocate to the shoots, which could be due to a lack of or low expression of amino acid permeases. These findings suggest that certain cultivars have a high capacity for Se-AA biosynthesis, translocation, and accumulation.

We propose that cultivar selection is an important strategy to obtain vegetable cultivars with a high capacity for Se-AA biosynthesis, translocation, and accumulation. However, conventional breeding strategies generally take a relatively long time. The PFALs could be considered as an ideal instrument to accelerate the breeding system. The PFALs allow the plant phenological period to be shortened by controlling important key factors, such as the lighting conditions, temperature, and nutrients supplied, to accelerate plant growth and development and to achieve the mature stage in a relatively short period. Indeed, five generations of short-day crops can be produced per year using PFALs (Jähne et al., 2020).

### Regulation of Total Se and Se-AAs in Vegetables by PFAL and Artificial Intelligence

The PFALs equipped with intelligent decision support systems have the potential to be a valuable instrument for producing vegetables with the desired Se contents and high proportions of Se-AAs. Machine learning algorithms based on artificial intelligence have been widely used in agriculture, for applications, such as the prediction of gene function (Mahood et al., 2020), crop yield (Yoosefzadeh-Najafabadi et al., 2021), and heavy metal contents (Shi et al., 2016). However, there are no examples of machine learning to study the regulation of health-promoting components in vegetables.

**Figure 5:**
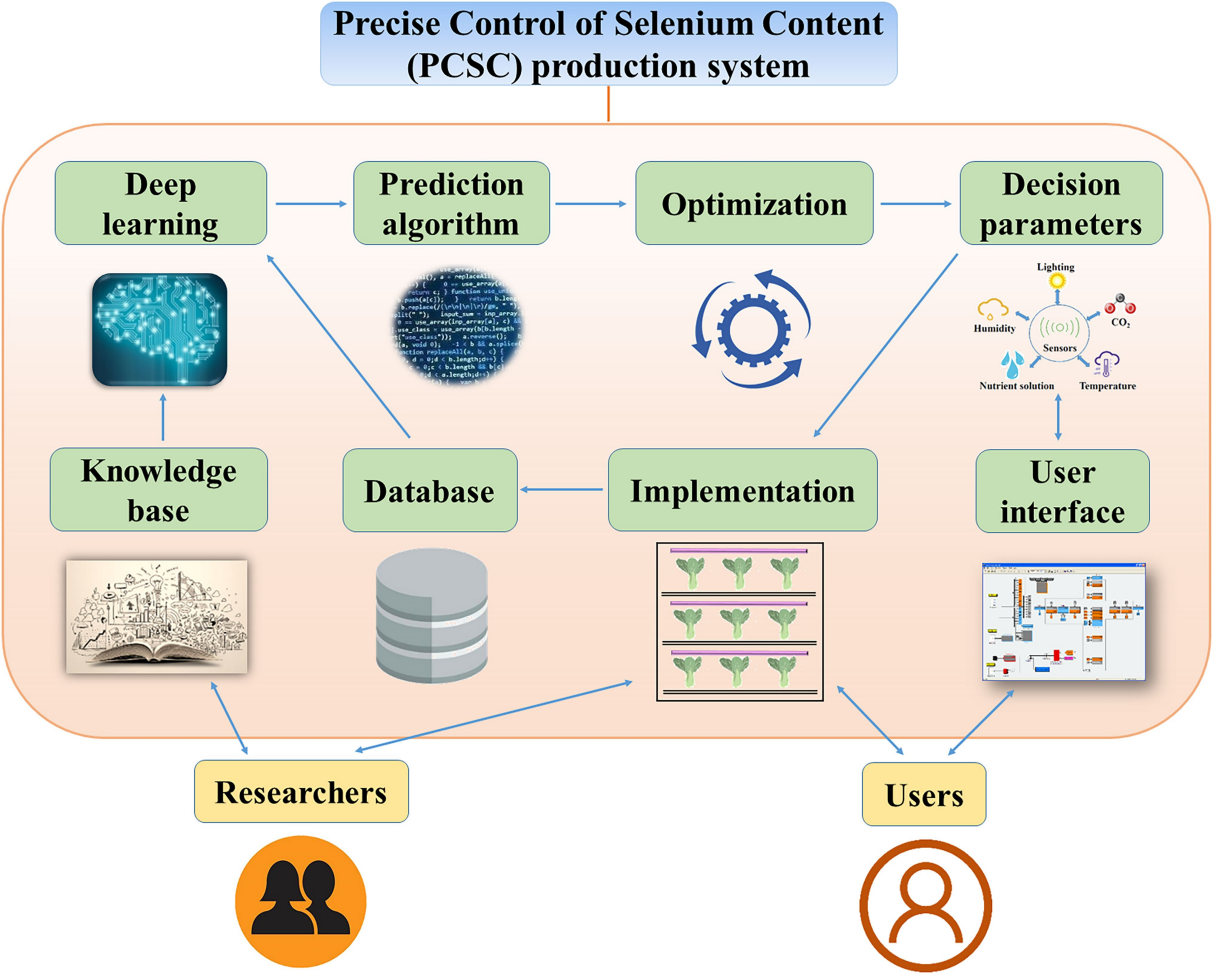
A Precise Control of Selenium Content (PCSC) production system for precise control of the forms and selenium contents in vegetables.

Here, we propose a Precise Control of Se Content (PCSC) production system to regulate the total Se and Se-AA contents of vegetables (Figure 5). To initiate this system, the researchers would define the treatments based on the desired Se content for the vegetables. Experimental data on the forms of Se, lighting conditions, humidity, temperature, and cultivar would be collected to establish a knowledge base. Next, the knowledge base would be utilized in machine deep learning. The decision parameters could be obtained by selecting an appropriate prediction algorithm according to the users’ objectives. However, to make more accurate decisions, an optimization model needs to be constructed. Then, the decision parameters would be tested in the PFAL. The results of the implementation would be collected in the database and would be subjected to further deep learning and prediction algorithm processes. After accumulation of vast data and repeated optimizations, the results would become increasingly accurate and therefore enable precise control of the total Se and Se-AA contents of vegetables. Although Se source, environmental condition, and plant species are known to affect the accumulation and regulation of the total Se and Se-AA contents of vegetables, detailed data and the precise mechanics remain vague. Therefore, further investigations are required to establish the proposed PCSC production system.

## Author Contributions

JH and XY created the hypothesis, objectives, outline the draft, and wrote the manuscript. ZW, LZ, JP, TH, BJ, and QY edited and added the discipline-specific feedback. All authors contributed to the article and approved the submitted version.

## Funding

This work was financially supported by the National Key Research and Development Program of China (no. 2020YFE0203600), Central Public-Interest Scientific Institution Basal Research Fund (no. Y2021XK04), and the Agricultural Science and Technology Innovation Program (34-IUA-01 and 34-IUA-03).

## Conflict of Interest

The authors declare that the research was conducted in the absence of any commercial or financial relationships that could be construed as a potential conflict of interest.

## Publisher’s Note

All claims expressed in this article are solely those of the authors and do not necessarily represent those of their affiliated organizations, or those of the publisher, the editors and the reviewers. Any product that may be evaluated in this article, or claim that may be made by its manufacturer, is not guaranteed or endorsed by the publisher.
